# Acute Motor Axonal Neuropathy Coexisting With Ankylosing Spondylitis: A Case‐Based Review

**DOI:** 10.1155/crii/5406702

**Published:** 2026-05-28

**Authors:** Yiğit Can Güldiken, Batool Achmar, Sema Kaymaz Tahra

**Affiliations:** ^1^ Department of Neurology, Faculty of Medicine, Canakkale Onsekiz Mart University, Canakkale, Türkiye, comu.edu.tr; ^2^ Faculty of Medicine, Bahcesehir University, Istanbul, Türkiye, bahcesehir.edu.tr; ^3^ Department of Rheumatology, Faculty of Medicine, Bahcesehir University, Istanbul, Türkiye, bahcesehir.edu.tr

**Keywords:** acute axonal motor neuropathy, ankylosing spondylitis, Guillain Barre syndrome, idiopathic autoimmune disorders

## Abstract

Guillain‐Barré syndrome (GBS) is a rare autoimmune disorder characterized by peripheral nerve involvement. Among the many kinds of GBS, acute motor axonal neuropathy (AMAN) syndrome has been shown to have distinct clinical characteristics and consequences. Meanwhile, ankylosing spondylitis (AS) is a long‐term inflammatory joint illness that mostly affects the sacroiliac joints and the spine; this disorder has been known to cause discomfort and stiffness, which may lead to decreased flexibility over time. Some studies have found a link between anti‐TNF α medication and GBS. GBS was commonly related to anti‐TNF‐α treatment for established AS, which explains the rare overlap of both diseases. In our case, nevertheless, both conditions occurred together without any confirmed therapeutic or infectious provocation. We describe a case of a 35‐year‐old painter hospitalized with paralysis of the lower and upper limbs but normal reflexes. Electromyography (EMG) demonstrated axonal motor neuropathy, and a lumbar puncture revealed high protein levels (42.2 mg/dL) and a normal cell count, all of which are indicators of GBS; hence, he was diagnosed with AMAN. Additional testing revealed human leukocyte antigen (HLA)‐B27 positive and bilateral sacroiliitis on magnetic resonance imaging (MRI), which is consistent with AS. The administration of plasma exchange (PLEX) treatment improved the bulbar function.

## 1. Introduction

Guillain‐Barré syndrome (GBS) is subdivided into many specific subtypes depending on the affected nerve fibers, major etiology of injury, and/or loss of sensations. Acute inflammatory demyelinating polyneuropathy (AIDP), the common form, is characterized by episodic polyneuropathy due to demyelination of the peripheral nerve with ascending muscle weakness and reduced muscle tone [1, 2].

Ankylosing spondylitis (AS) is a chronic inflammatory disease mainly affecting the spine and sacroiliac joints [3]. It typically onsets before the age of 40 and is more prevalent in males. It may also manifest peripherally as uveitis, arthritis, enthesitis, psoriasis, aortic root, and gut inflammation [4, 5].

Its onset predisposes several causative factors, which are both hereditary as well as environmental. The strongest genetic association is with the human leukocyte antigen (HLA)‐B27 gene, which can be seen in around 70%–90% of AS patients. Some environmental factors are believed to play a role, especially bacterial infections. For instance, the infection of *Klebsiella pneumoniae* and other bacteria in the gut has been associated with the onset and development of AS [3, 6, 7].

The AS/acute motor axonal neuropathy (AMAN) overlap presents a rare complexity, which is characterized by the coexistence of two autoimmune conditions. The latter primarily affects the motor nerves, specifically the axons of the motor neurons [1].

Herein, we present a case report of a patient suffering from AS alongside AMAN. The absence of clinical and laboratory evidence of prior infection and lack of medication history prompts the consideration of an idiopathic autoimmune etiology underlying the observed neurological manifestations.

## 2. Case Report

A 35‐year‐old male presented to the outpatient clinic in January 2024. The patient, who worked as a painter was healthy until August 2023, when walking problems, difficulties while climbing the stairs, and paraparesis started. He described having swelling in his left knee and back pain, particularly noted during transitions from lying to standing positions.

In November 2023, he had difficulty even in going to the bathroom and was not able to walk for the last month. He suffered from muscle twitching in his whole body, accompanied by weakness in the hands and arms for the last 5–6 months. Additionally, he lost 20 kg and suffered from a loss of appetite during the last 6 months. Shortness of breath was present for 1 month, accompanied by stomachache, dry mouth, and a dry cough. There was no history of diarrhea or upper respiratory tract infection prior to admission.

During the physical examination, it was noticed that he had a passive posture and was cachectic. For muscle strength, in the lower right extremity, there was 3/5 power loss, and in the lower left extremity, 2/5 power loss. Knee and plantar reflexes were normal, and Hoffmann’s sign was negative. He had severe quadriceps atrophy. Additionally, he complained about shortness of breath and tightness in the chest. When examined, it was noticed that accessory respiratory muscles also participated in breathing.

During the rheumatologic assessment, he did not describe any active peripheral joint complaints. The right gluteal pain was present for 5 months, especially when sitting and getting up. He had a history of joint swelling in the left knee 5 years ago. During the examination, there was no peripheral arthritis. The sacral thrust test was positive in the right sacroiliac joint. In the detailed anamnesis, he had no uveitis, psoriasis, inflammatory bowel disease, enthesitis, or thrombosis history. Also, he did not have oral aphthosis, genital ulcers, rash, eye dryness, or Raynaud’s complaints.

Electromyography (EMG) test showed decreased compound muscle action potential (CMAP) responses in upper extremities, and in lower extremities, CMAP could not be obtained, which could be due to extensive muscle wasting. In needle EMG examination, extremely chronic neurogenic motor unit potentials (MUPs) changes and normal MUPs could not be obtained in the bilateral proximals of the upper and lower extremities (much more evident in the lower extremities) (Table 1). Intense fasciculations in spontaneous activity were observed in the upper and lower extremities. These findings were evaluated as compatible with common motor horn involvement, which is much more specific in bilateral upper and lower extremities, and lower extremities’ proximals. Accordingly, axonal polyneuropathy, which affects the peripheral nervous system, and paraneoplastic syndrome were suspected in the patient. Spinal muscular atrophy (SMA) genetic test was performed, and the result was negative. Also, due to the rapid progression of motor activity loss, amyotrophic lateral sclerosis (ALS) disease suspicion was dropped. Total‐body positron emission tomography (PET) scanning showed no malignant masses. The patient was diagnosed with AMAN, which is a variant of GBS. Later, high titers of anti‐GD1a and anti‐GQ1b IgG antibodies confirmed the diagnosis.

**Table 1:** EMG results demonstrating AMAN.

Motor nerve conduction studies				
Stimulation site	Latency (ms)	Amplitude (μV)	Velocity (m/s)	Distance (cm)
R ulnar: ADM				
Wrist	2.5	6.1		80
Elbow	7.5	4.4	50.0	250
Arm	10.0	4.1	40.0	100
L ulnar: ADM				
Wrist	2.8	5.3		80
Elbow	8.6	5.1	48.7	280
Arm	10.5	4.6	47.4	90
R median: APB				
Wrist	3.2	3.3		
Elbow	8.2	2.4	52.0	260
L median: APB				
Wrist	3.5	3.5		
Elbow	8.3	2.7	54.7	260
R tibial: AH				
		0		
L tibial: AH				
Medial malleolus	6.7	0.8		70
Popliteal fossa	18.6	0.40	36.7	440
R common peroneal: EDB				
Sole of the foot		0		
L common peroneal: EDB				
Sole of the foot		0		

Sensory nerve conduction studies				
Stimulation site	Latency (ms)	Amplitude (μV)	Velocity (m/s)	Distance (cm)
R, n. median, II digit				
Wrist	2.7	20.5	55.6	
L, n. median, II digit				
Wrist	2.8	21.3	54.5	
R, n. ulnaris, V digit				
Wrist	2.5	20.2	51.0	
L, n. ulnaris, V digit				
Wrist	2.4	20.6	52.1	
R, sural, lateral malleolus				
Calf	1.9	14.7	60.5	
L, sural, lateral malleolus				
Calf	2.0	15.4	61.5	

QEMG				
Fasciculation	MUP amplitude	MUP duration	Muscle pattern	
R axillary: deltoid				
No	Normal	Normal	Normal	
L axillary: deltoid				
++	Greatly increased	Significantly increased	Neurogenic	
L musculocutaneous: BB				
+++	Greatly increased	Significantly increased	Neurogenic	
R median: APB				
++	Decreased	Increased	Neurogenic	
R femoral: RF				
++	Sharply increased	Increased	Neurogenic	
R tibial: gastrocnemius				
No	No	No	Neurogenic	
R peroneal: TA				
++	No	No	Neurogenic	
L peroneal: TA				
No	No	No	Neurogenic	

*Note:* The nerve conduction study showed axonal motor neuropathy in both upper and lower extremities, indicated by reduced amplitudes in motor nerves. However, it was more prominent in the lower extremities, as no amplitudes could be obtained in the right tibial nerve and both right and left peroneal nerves. In contrast, the sensory conduction study showed normal values.

Abbreviations: ADM, abductor digiti minimi; AH, abductor hallucis; APB, abductor pollicis brevis; BB, biceps brachii; EDB, extensor digitorum brevis; RF, rectus femoris; TA, tibialis anterior.

Dorsal magnetic resonance imaging (MRI) showed dorsal kyphosis, significant degenerative osteoarthritic changes in the upper dorsal region, and degenerative disc disease. In lumbar MRI, it was noticed that there were median herniations in L4‐5 and L5‐S1 discs, narrowing in the anterior epidural spaces, dural sac compressions, and bilateral sacroiliitis. HLA‐B27 test was positive.

Based on the MRI results and the ASAS classification criteria for axial spondyloarthritis, the diagnosis of AS was therefore established [8]. Diclofenac was given for the treatment.

Meanwhile, cervical and brain MRI were performed, and they were normal.

Laboratory examinations revealed high C‐reactive protein levels (CRP) (23.78 mg/L), indicating a potential inflammatory response. Blood cell count, serum electrolyte levels, liver, and renal function test results were normal. Antinuclear antibody (ANA) testing was negative. The extractable nuclear antigen test showed low‐titer anti‐dsDNA positivity, which was assessed as clinically insignificant.

Thoracic MRI was performed due to chest symptoms and showed a nodule at the apex of the upper lobe of the right lung. Also, lumbar puncture revealed high glucose (71 mg/dL), high albumin (0.3 g/dL), and high protein (42.2 mg/dL) levels. These elevated cerebrospinal fluid levels could suggest a disruption in the blood‐CSF barrier, which may be associated with the patient’s AMAN diagnosis.

In the following 7 days of admission, the patient started experiencing problems in swallowing. Ten days after admission, a serum was administered to the patient for vitamins and minerals supplementation due to low oral intake caused by dysphagia.

Thirteen days following admission, plasma exchange (PLEX) therapy was initiated because it had been proven to be more effective in AMAN [9] and also due to the bulbar involvement in the patient. Three days after the initiation of PLEX, thorax CT was taken, and it showed pneumonic infiltration in the lower lobe of the right lung, so the patient was started on piperacillin‐tazobactam. CRP was 177–208 mg/L, sedimentation rate was 76 mm/h, and INR was 1.23. Aspiration pneumonia was primarily considered in the patient.

On the 18th day following admission, complaints about constipation arose. Bisacodyl was prescribed. Also, it was noticed that the swallowing reflex was greatly improved to a level where the patient could start oral intake. However, no documented improvement in limb muscle strength was observed. Physical therapy was initiated, and the patient was scheduled to be discharged within the next few days.

## 3. Literature Review

Using the PubMed and Google Scholar search engines with the keywords “Guillain Barre syndrome” or “Miller Fisher syndrome” and “Ankylosing spondylitis” we found seven patients with AS and GBS [10–16]. Including the case we described, the mean age at diagnosis of GBS was 45 (with a range of 24–76), and five out of eight patients (62%) were men. The most common presenting symptoms of GBS were areflexia in knee reflexes and weakness in lower and upper limbs. However, our patient had bulbar involvement in addition to these symptoms. Regarding treatment, while most cases used intravenous immunoglobulin (IVIG) in their treatment regimen, our patient received only PLEX treatment and showed improvement in bulbar function. A summary of these cases is shown in Table 2.

**Table 2:** Literature review.

Author publication year	Gender	Age at diagnosis of GBS (year)	SpA subtype	Disease duration of SpA (year)	Clinical presentation of GBS	Probable Triggers of GBS	Treatment for GBS	Outcome
Psarelis et al. 2017 [10]	Male	50	AS	5	Weakness in upper and lower limbs, muscle power 4/5 biceps in both arms, 3/5 quadriceps in both legs, absence of tendon reflexes	Etanercept treatment	IVIG, PLEX secukinumab	Complete resolution
Reeker et al. 1999 [11]	Male	76	AS	NA	NA	NA	NA	Complete resolution
Uğurlu et al. 2014 [12]	Male	69	AS	30	Lower extremity weakness, hypotonia and hyporeflexia on the right and areflexia on the left lower extremity	Adalimumab treatment	IVIG	Gradual improvement in lower extremity muscle strength
Amine et al. 2009 [13]	Female	47	AS	~2 years (from 2005 to May 2007)	Paraesthesia of hands and lower limbs, reduced power in lower limbs, reduced knee and ankle reflexes, mute plantar responses	Infliximab treatment	IVCS, IVIG	Complete resolution
Shobha et al. 2019 [14]	Male	24	AS	NA	Weakness in both lower limbs with buckling, power 3/5 in both lower limbs, sluggish reflexes	Etanercept biosimilar treatment	High dose dexamethasone followed by 5 days of IVIG	Complete resolution
Kim et al. 2018 [15]	Female	30	AS	21	Limb ataxia, spasticity, acute ophthalmoplegia, hyperreflexia, disturbed consciousness (drowsiness)	Adalimumab treatment	High‐dose methylprednisolone, IVIG, rituximab	Rapid improvement in mental status and recovery from ophthalmoplegia following treatment with rituximab
Lachhab et al. 2025 [16]	Female	28	AS	NA	Paresthesia in both lower limbs and the right upper limb, flaccid tetraplegia, altered consciousness, severe respiratory distress	NA	PLEX	Motor deficits (upper limbs 3/5, lower limbs 2/5)

*Note:* Literature review of patients with coexisting AMAN and ankylosing spondylitis.

Abbreviations: IVCS, intravenous corticosteroids; IVIG, intravenous immunoglobulin; PLEX, plasma exchange.

## 4. Discussion

In AMAN and AMSAN, examination of motor and sensory nerve conduction studies shows that there is degeneration of the axon, not demyelination [1, 2]. Meanwhile, distal CMAP amplitudes are reduced by more than 10% below the lower limit of normal. When these electrophysiological abnormalities hit only motor fibers without involving the sensory components, AMAN is diagnosed. However, if additional deficits due to involvement of sensory fibers take place, such a condition is termed AMSAN [17]. Miller Fischer syndrome is a rare variant of GBS where the clinical triad namely ophthalmoplegia, ataxia, and areflexia occurs without much weakness [1].

The most common cause of GBS is an autoimmune response triggered by either viral or bacterial infection. *Campylobacter* infectious disease was reported to be the cause of 30%–40% of GBS cases [18].

The clinical presentation of AMAN often mimics other neurological conditions, posing diagnostic challenges. In our case, the patient initially exhibited normal knee reflex and lower limb weakness. The presence of intense fasciculations in the whole body, which is a hallmark of ALS [19], altogether with the other symptoms was indicative of anterior horn disease, prompting considerations of ALS.

The typical age when patients first present with ALS is between 55 and 75 years [20]. On the other hand, the onset of AMAN peaks in both young and old people [2]. In addition, both diseases can occur at any age [2, 20]. AMAN disease has a very rapid progression, and patients usually reach their weakest point within 4 weeks [21]. ALS usually presents with initial symptoms in a single limb on one side of the body. It then moves to the matching limb on the other side, the upper limb on the same side, and eventually, the remaining upper limb on the opposite side. This is a stepwise deterioration of the motor neuron, which is steady and uniform in its propagation, ending in bulbar weakness, which affects speech, swallowing, and respiratory functions.

As to the prognoses, most patients with ALS progress to a state of great motor dysfunction with poor outcomes, often succumbing to respiratory insufficiency within an average of 3–5 years. ALS is currently untreatable and usually results in death [22]. AMAN, on the other hand, has a favorable outcome because it is usually treatable. Most patients with AMAN have significant improvement after a span of several months, although the more severe cases may have a permanent disability and, in rare instances, death [23].

Upper motor neuron involvement in ALS can be demonstrated by Babinski and Hoffman signs, spasticity, and excessive deep tendon reflex, DTR [24]. These signs were not present in our patient. Our patient showed normal DTR, which is rarely seen in GBS [25] and is suggestive of AMAN [26]. In addition, dysphagia and respiratory problems resolved after PLEX therapy, providing strong evidence refuting the presence of ALS [27].

The sudden widespread muscle twitching, typical of ALS [24], has rarely been observed in AMAN patients [28]. However, this may suggest the possibility of both disorders being related since they both affect peripheral motor neurons [1, 24]. There was one reported case of a patient with a history of AMAN who later went on to develop ALS, suggesting that the two diseases may well in fact be related, irrespective of the etiology [29].

In this case of AMAN, the cause of fasciculations could be ascribed to occupational hazards since the patient had worked as a painter. There is evidence that chronic exposure to organic solvents, the most notorious of which is toluene, and heavy metals such as lead and chromium used in automotive paints are associated with neuromuscular disorders. One of the studies in Thailand’s car body‐restoration business pointed out the neurotoxic chemicals present in the paints sprayed during work are responsible for some severe injuries and even more severe neurological symptoms [30]. Regrettably, we could not send the patients’ blood for toxicology testing for search of curative measures of these substances; hence, the presence of an environmental factor could not be proven.

In our case, the intact sensory nerve function observed in the sural nerve during EMG and the absence of mutations associated with SMA further shifted the diagnostic trajectory towards AMAN.

AMAN can be associated with autonomic dysfunction. Mostly, the sudomotor hypofunction is seen in patients with severe neurological deficits [31, 32].

In our patient, the clinical manifestations shifted over the course of the illness. He experienced right gluteal discomfort for 5–6 months and left knee swelling for around 5 years, which are symptoms typically linked to AS [3–8, 16], and might lead to ongoing functional impairment. This clinical picture was succeeded by increasing walking difficulties, weakness in the limbs, and weight loss over the subsequent months, ending with a rapid neurological decline in the last month resulting in total inability to walk. Hence, he required immediate hospitalization.

AS symptoms include morning stiffness, peripheral joint involvement, and persistent inflammatory pain in the back or gluteal region [3–8, 16]. These symptoms can culminate in decreased mobility and an irregular gait. As for AS symptoms, they typically develop slowly over many years, and early complaints may go undiagnosed or be mistaken for mechanical or nonspecific musculoskeletal issues for a long time [4, 6–8]. On the other hand, AMAN typically develops over a period of days to weeks [21]. It is defined by a quick progressive motor weakness, areflexia, and occasionally bulbar involvement [1]. Despite these different pathophysiological conditions, both diseases can elicit symptoms such as weakness, fatigue, decreased functioning, and difficulty with walking, which may obscure the underlying cause in a particular patient [1, 3–8, 16].

Given this clinical picture, we suggest that AS may have occurred prior to the manifestation of AMAN, thereby attributing the abrupt neurological deterioration to the occurrence of AMAN. Nonetheless, we emphasize that this temporal association cannot be conclusive, as the clinical diagnosis of AMAN by neurological examination and testing preceded that of AS during rheumatologic consultation. As a result, the sequence of diagnosis in our report does not correspond to the disease’s validated timeframe.

We also thoroughly assessed the potential of chronic inflammatory demyelinating polyneuropathy (CIDP). Nevertheless, various characteristics countered this diagnosis, such as the lack of sensory involvement, the absence of demyelinating features or conduction block in electrophysiological tests, positivity of antiganglioside antibodies (anti‐GD1a and anti‐GQ1b), and the clinical improvement after PLEX treatment.

With the diagnosis of AMAN confirmed, the probable relationship between AS and axonal GBS becomes the primary focus of interest and the novel part of the current case. To our knowledge, our case is the first to present the coincidence of GBS and AS without any confirmed interference to their etiology.

AS is significantly influenced by genetic and environmental factors [4]. Genetic predisposition for AS was noted by the occurrence of the HLA‐B27 haplotype in affected individuals [7, 33].

Our patient demonstrated a susceptibility to AS development due to the presence of a positive HLA‐B27 test. However, environmental factors such as infection or vaccination may also play a significant role in GBS [34] and AS [4] causation and progression. Therefore, thorough investigation is necessary for patients presenting with such comorbidities.

Previous literature scarcely reported such an association. With few cases reporting neurological complications in AS patients due to etanercept, adalimumab, and infliximab treatment leading to the development of GBS [10–16]. Although the exact mechanism by which these medications triggered GBS is not fully understood, it is important to note that such complication risk is relatively low. In contrast to most previously reported AS–GBS cases [10–16], our patient had not received any biologic therapy prior to the onset of AMAN, making a medication‐related etiology unlikely. However, the patient’s occupational history as a painter raises the possibility that environmental or toxic exposure may have acted as a potential trigger in a susceptible host, although a causal relationship cannot be established. Therefore, this case may represent either a coincidental coexistence of AS and AMAN or a manifestation precipitated by nontherapeutic factors.

In terms of treatment considerations, previous data described in Table 2 show that IVIG was used to treat most AS‐GBS cases [10, 12–15], whereas PLEX was used as the initial therapy for only a small percentage of cases [10, 16], including our patient. After PLEX, our patient showed improvement in bulbar function. Our treatment mainly focused on AMAN‐directed immunotherapy in addition to supportive symptomatic care for AS, as our patient had not received disease‐modifying medication for AS, unlike other cases in which neuropathy developed after biologic therapy [10, 12–15].

In addition, some cases reported the coincidence of AS and multiple sclerosis (MS), which is a disease that affects the central nervous system. In some cases, MS was induced by the anti‐TNFα treatment of AS [35], while in others the causation remained obscure [36].

While AS‐related neurological involvement is rare, in a study done on 1472 Brazilian patients diagnosed with spondylarthritis, only 13 (0.9%) had neurological involvement, which was mostly related to atlantoaxial subluxation and cauda equina syndrome [37].

Dysphagia is a frequently seen symptom in patients with AMAN [1]. In a retrospective study done on 88 patients with AMAN, symptoms of upper respiratory infection were seen in 20% of the patients [38]. Our patient had suffered aspiration pneumonia twice during his stay in the hospital, which negatively affected his course of rehabilitation severely.

## 5. Conclusion

In summary, this case shows the complexity associated with controlling the coexistence of rheumatological and neurologic disorders. It also highlights the wide spectrum of autoimmune disease and its relationship with the neurological conditions.

This case demonstrates the need for multidisciplinary assessment and care provision in patients with multiple comorbidities, because even standard treatments are potentially fatal in these situations. Hence, the presence of practitioners from several disciplines may improve patient’s outcomes, minimize complications, and optimize care quality.

## Funding

No funding was received for this manuscript.

## Conflicts of Interest

The authors declare no conflicts of interest.

## Data Availability

The data that support the findings of this study are available upon request from the corresponding author. The data are not publicly available due to privacy or ethical restrictions.
